# A qualitative assessment of the perceived risks of electronic cigarette and hookah use in pregnancy

**DOI:** 10.1186/s12889-015-2586-4

**Published:** 2015-12-21

**Authors:** Maike K. Kahr, Shannon Padgett, Cindy D. Shope, Emily N. Griffin, Susan S. Xie, Pablo J. Gonzalez, Judy Levison, Joan Mastrobattista, Adi R. Abramovici, Thomas F. Northrup, Angela L. Stotts, Kjersti M. Aagaard, Melissa A. Suter

**Affiliations:** Department of Obstetrics and Gynecology, Division of Maternal Fetal Medicine, Baylor College of Medicine, 1 Baylor Plaza, Houston, TX 77030 USA; Department of Obstetrics and Gynecology, University of Texas Medical School at Houston, 6431 Fannin Street, Houston, TX 77030 USA; Department of Family and Community Medicine, University of Texas Health Science Center at Houston, 6431 Fannin Street, JJL 324, Houston, TX 77030 USA

**Keywords:** Electronic cigarettes, E-cigarettes, E-cig, Hookah, Focus group, Pregnancy

## Abstract

**Background:**

Studies reveal that electronic cigarette (e-cigarette) and hookah use are increasing among adolescents and young adults. However, the long-term health effects are unknown, especially with regards to pregnancy. Because of the increased use in women of reproductive age, and the unknown long-term health risks, our primary objectives were to determine the perceived risks of e-cigarette and hookah use in pregnancy, and learn common colloquial terms associated with e-cigarettes. Furthermore, we sought to determine if there is a stigma associated with e-cigarette use in pregnancy.

**Methods:**

Eleven focus groups including 87 participants were conducted immediately following regularly scheduled CenteringPregnancy® prenatal care with women at three different clinics in the greater Houston area. A minimum of two facilitators led the groups, using ten lead-in prompts, with Spanish translation as necessary. Facilitators took notes which were compared immediately following each group discussion and each group was audio recorded and transcribed. Three facilitators utilized NVivo 9.0 software to organize the transcribed data into nodes to identify major themes. To increase rigor, transcripts were further analyzed by two obstetricians who were instructed to find the major themes.

**Results:**

Analyses revealed contradicting themes concerning e-cigarette use. In general, e-cigarettes were perceived as safer alternatives to regular tobacco cigarettes, especially if used as smoking cessation devices. A major theme is that use in pregnancy is harmful to the fetus. However, it was perceived that use for smoking cessation in pregnancy may have fewer side effects. We found that a common term for e-cigarettes is “Blu.” In our discussion of hookah use, participants perceived use as popular among teenagers and that use in pregnancy is dangerous for the fetus.

**Conclusions:**

Although a strong theme emerged against hookah use, we found contradicting themes in our discussions on e-cigarette use in pregnancy. It is possible that e-cigarette use will not carry the same stigma as regular cigarette smoking in pregnancy. In addition, the impression of e-cigarettes as a healthier alternative to smoking may influence use in pregnancy. Clinicians need to be prepared for questions of e-cigarette safety and efficacy as smoking cessation devices from their pregnant patients who smoke, and women who smoke and are planning to become pregnant.

## Background

Use of electronic cigarettes (e-cigarette) and hookah is on the rise among adolescents [1]. E-cigarette safety is a topic under heated debate, with proponents highlighting the potential benefit as a harm reduction product for current smokers, while public health officials express concern about the lack of data on long-term health risks [2]. Recent data show that e-cigarette use is becoming more acceptable among middle and high school students [3], as the amount of these students who reported ever use of e-cigarettes doubled between 2011 and 2012 and continues to rise [4]. Similarly, hookah use is increasing in high school students, where one study revealed that as many as 20 % of high school seniors have tried hookah [5, 6].

Considering the rise of e-cigarette and hookah use among young people, including females of reproductive age, questions emerge regarding the safety of use during pregnancy, and the immediate and long-term health risks for both mother and fetus. In other words, do pregnant and reproductive aged women consider e-cigarettes safer than, equivalent to, or higher risk than combustible tobacco cigarettes? Is this concordant or discordant from current recommendations and existing knowledge? By addressing these gaps in consumers’ knowledge can we enable informed decision regarding use, and potentially prevent long term and multigenerational exposures?

E-cigarettes deliver liquid nicotine, amongst other ingredients, as an aerosol produced by heating and vaporizing the liquid components through a battery charged atomizer. The composition of the liquid component varies and is often composed of flavoring substances, propylene glycol and glycerin [7]. Scientific publications vary in the levels of carcinogens and harmful substances found in these devices due to variation among products made by different companies [8]. However, e-cigarettes are marketed as smoking cessation devices [7] and as better alternatives to regular cigarettes [9]. This perception of harm reduction in comparison to regular cigarettes may be prevalent due to the lack of FDA regulation on e-cigarette advertising.

While e-cigarettes are marketed as smoking cessation devices [10, 11], there are few studies supporting this claim [1, 12]. Due to known adverse effects of smoking on the fetus, pregnant women are counseled on smoking cessation. Presently there are no clear recommendations as to whether nicotine replacement therapy in pregnancy substantially increases the likelihood of successful cessation. Use of the nicotine patch as a method to quit smoking during pregnancy has not been shown to be effective, possibly due to higher metabolism of nicotine in pregnancy or lower adherence [13–16]. Recent data from a randomized controlled trial reveal that infants born to smoking mothers who used a nicotine containing patch for smoking cessation during pregnancy were less likely to have impaired development compared to those whose mothers received a placebo patch [17]. The potential for pregnant women to utilize e-cigarettes due to the claims of their efficacy as cessation devices is a strong possibility. However, their efficacy amongst gravidae is unknown at present.

Hookah, also called “water pipe,” delivers tobacco smoke with various contents to the smoker [18, 19]. It consists of a head, body, water bowl, hose and mouthpiece and can be filled with tobacco of different flavors, which appeals to adolescent users [18, 20]. A piece of coal is lit and placed on top of the head. By inhaling through the mouthpiece, the tobacco smoke enters the water bowl and reaches the mouthpiece through the hose [21]. Research suggests that smoking hookah delivers similar amounts of carbon monoxide as regular cigarette smoking [22, 23]. Further contents of hookah smoke comprise heavy metals such as arsenic, chromium and lead, as well as nicotine and tar [21]. The rise in popularity of hookah bars reflects the increased prevalence of hookah use [24].

For decades the adverse effects of smoking during pregnancy have been widely studied and reported [25–27]. Yet our knowledge of the effects of prenatal nicotine use (rather than as a component of combustible tobacco smoke) mostly comes from studies using animal models. Adverse effects include altered offspring lung development, metabolism and neurobiology [1]. Risks for hookah use in pregnancy have also been reported [28]. Not only does hookah smoking during the first trimester of pregnancy almost triple the risk of a low birth weight baby, these neonates also exhibited lower APGAR scores and were more likely to suffer from pulmonary issues [28]. Hence the effects of hookah smoke exposure should be taken seriously.

The health effects of electronic cigarettes, especially with regards to pregnancy, are still poorly understood [29–31]. With the rising popularity of e-cigarettes within the adolescent and young adult populations, it was our goal to determine the perception of risks of e-cigarette and hookah use in pregnancy. For this study we conducted focus groups with pregnant women who were participating in the CenteringPregnancy® model of prenatal care. Our primary objectives were to determine the perceived risks of e-cigarette and hookah use during pregnancy, as well as to determine if there is a stigma associated with e-cigarette use while pregnant. A secondary objective was to determine common terms and colloquialisms for e-cigarettes and their use.

## Methods

### Participants

This work was done in accordance with an approved IRB protocol from Baylor College of Medicine (H-34725). This work adheres to RATS guidelines for reporting the results of qualitative research [32]. Eleven focus groups were conducted at three clinics in the greater Houston area with a total of 87 pregnant women participating. A minimum of three focus groups were conducted at each location to minimize bias based on clinic demographics. Inclusion criteria included gravidae currently enrolled in the CenteringPregnancy® prenatal care group who had English or Spanish proficiency and were able to provide informed consent. Through the CenteringPregnancy® model, pregnant women meet once to twice a month for routine clinical prenatal care and are able to share pregnancy experiences with women who have similar due dates [33–35]. At our clinics, women are offered either the standard of care (ie to meet with their physician or midwife one-on-one) or to participate in the CenteringPregnancy® groups. Due to the nature of focus group research, having a group of 6-12 people is ideal [36], making the CenteringPregnancy® groups suited for this research. Women were informed about the research project at the meeting prior to our focus group, and it was explained that their participation is voluntary and not part of their prenatal care. The focus groups were conducted after their regularly scheduled meeting so women who did not wish to participate were able to leave before commencement of the focus group.

The groups were conducted at the Cullen Teen Health Clinic, Vallbona Health Clinic and The Center for Children and Women (Table 1). At the Cullen Clinic the majority (57 %) of pregnant women who participate are African American, and 40 % are Hispanic. At Vallbona Health Clinic the overall composition of the CenteringPregnancy® program are 6 % African American, 6 % Caucasian, 82 % Hispanic and 6 % Other. The groups at The Center for Children and Women are 68 % Hispanic, 23 % African American, 6 % Caucasian and 2 % Other. No identifying or demographic information was collected from the participants in accordance with our approved IRB.

**Table 1:** Race/ ethnicity of women participating in CenteringPregnancy®

Clinic	Race/ ethnicity	Participants
Cullen Teen Health Clinic	African American (57%)	3 focus groups,
	Hispanic (40%)	24 participants
	Other (3%)	
Vallbona Health Clinic	Hispanic (82%)	3 focus groups,
	African American (6%)	34 participants
	Caucasian (6%)	
	Other (6%)	
The Center for Children and Women (Greenspoint)	Hispanic (68%)	5 focus groups,
	African American (23%)	29 participants
	Caucasian (6%)	
	Other (2%)	

### Procedure

For the 11 focus groups, we utilized established CenteringPregnancy® prenatal care groups. Ten semi-structured, focused, lead-in prompts were used as a framework for discussions (Table 2). These prompts were designed to understand the women’s perceived risk of e-cigarette and hookah use in pregnancy, to determine alternate terms used when discussing e-cigarettes and to determine if there is a potential stigma associated with e-cigarette use in pregnancy. A minimum of two study facilitators, as well as a Spanish translator (as needed), attended each group. Upon entering the room the facilitators introduced themselves, explained the purpose of the study, and explained that the discussion would be audio recorded. The facilitators discussed that no names should be used during the focus group to protect anonymity. Informed consent was obtained in writing from all participants prior to starting the discussion. All participants received a $10 gift card for completing the study.

**Table 2:** Lead in prompts for focus group discussion

1. Tell me what you know about electronic cigarettes.	
2. What words or terms have you heard used to describe electronic cigarettes?	
3. Where have you seen electronic cigarettes advertised or sold?	
4. How do electronic cigarettes compare to regular cigarettes?	
5. What would you say to a pregnant friend who is using electronic cigarettes?	
6. What would you say to a pregnant friend who is using electronic cigarettes to quit smoking traditional cigarettes?	
7. What do you think if you see a pregnant women smoking traditional cigarettes?	
8. What would you think if you saw a pregnant woman using an electronic cigarette?	
9. Tell me what you know about hookah.	
10. What would you say to a pregnant friend who is using hookah?	

All facilitators and translators are experienced researchers in the field of OB/GYN. Our team was advised by two behavioral psychologists (T. Northrup and A. Stotts) and two obstetricians (J. Levison and K. Aagaard) with expertise in focus group research. Analysis was performed under the direction of T. Northrup and A. Stotts.

Each focus group lasted approximately 20 min including introduction and obtaining informed consent. Questions were asked in English, and translated into Spanish as necessary. Answers in Spanish were translated into English as necessary. Facilitators transcribed the responses in writing and compared their written reports at the end of each group discussion. Focus groups were conducted until saturation was achieved and no new data were emerging as determined by a lack of additional themes from the final two focus groups. Any questions the participants had concerning e-cigarettes or hookah were answered upon completion of the focus group in order to avoid potential bias.

### Data analysis

All audio recordings were transcribed by individuals proficient in English and Spanish. NVivo 9 software (QSR International, Burlington, MA) was used for line-by-line coding of the transcribed audio recordings. Data coding and interpretation was performed by three independent facilitators. Due to the large number of study participants, each subject was not assigned a code number. Instead, data were coded into nodes (a node being defined as an overarching content area) according to the lead-in prompts. Each facilitator independently searched for themes (a theme being defined as an interpretive narrative which often stems from or connects nodes) which emerged from the focus groups [37, 38]. The three facilitators discussed their interpretation of both major and minor themes, and only ones which were unanimously agreed upon were considered as themes of the focus group discussions. Major themes were defined as the most common theme brought up in discussion for the specific lead-in prompt; while the minor themes were the second most common theme.

To increase rigor in our analysis, transcripts were given to two obstetricians who had not attended the focus group discussions and who were instructed to find the themes which emerged throughout the focus groups. These obstetricians have experience with nicotine research in pregnancy as well as focus group analyses. Their analysis was completely separate and blinded from the analyses of the three facilitators. Only themes which were unanimously determined are reported herein.

## Results

Analyses of transcripts from all focus groups revealed both major and minor themes which emerged from our discussion of electronic cigarettes and hookah use in pregnancy (Table 3).

**Table 3:** Summary of beliefs and attitudes of subjects emerging from focus group discussions

Nodes	Major Themes	Minor Themes
Comparison of e-cigarettes to regular cigarettes	1. E-cigarettes are a safer and healthier alternative to regular cigarettes	1. Lack of secondhand smoke 2. Contain vapor (rather than smoke) 3. Not as many chemicals 4. Can be used indoors
Electronic cigarette use in pregnancy	1. E-cigarettes are not safe in pregnancy and are likely harmful to the fetus 2. E-cigarettes may be as bad as regular cigarettes during pregnancy	1. Rather than using e-cigarettes to quit smoking while pregnant, mom’s should either just quit or use the patch or gum 2. Switching to e-cigarettes to quit smoking may have fewer side effects.
Stigma for e-cigarette use in pregnancy	1. There are risks for e-cigarette use in pregnancy 2. A mother who uses e-cigarettes in pregnancy is not taking care of her baby.	1. Because there are fewer side effects of e-cigarettes, it may not be as bad as smoking.
Terminology for e-cigarettes	1. Blu	1. E-cigs 2. Vaporizors 3. Smokeless or smokefree
Hookah knowledge	1. Popular with teenagers 2. Hookah bars are common	1. Tobacco comes in many flavors 2. Hookah can be seen on TV
Hookah use during pregnancy	1. Hookah in pregnancy is dangerous for mother and baby.	

### Knowledge of electronic cigarettes

Focus group discussions started with the prompt, “Tell me what you know about electronic cigarettes.” This allowed assessment of the baseline knowledge of study participants. While there was generally one person in each group who had not heard of them, participants were overall familiar of e-cigarettes with an awareness that they (1) were sold in different colors and flavors, (2) contained nicotine, (3) may be used as a smoking cessation device, and (4) can be addictive. Participants were also asked where they had seen e-cigarettes advertised and sold. The responses revealed that they had seen them sold in gas stations and at the mall, and advertised on television and via the internet. The following are representative of overall participant responses:

— *“I guess you smoke them when you don’t want to smoke cigarettes—like regular cigarettes.”*
— *“I’ve heard that you can get addicted.”*
— *“Well it’s because they’ve got nicotine in them.”*
— *“There’s different flavors.”*

### Comparison of electronic cigarettes to traditional tobacco cigarettes

Further prompts were used to understand how participants perceived e-cigarettes, especially in comparing them to traditional tobacco cigarettes. They were asked, “How do electronic cigarettes compare to regular cigarettes?” One major theme that emerged is that women believed e-cigarettes to be a healthier and safer alternative to smoking combustible tobacco cigarettes. Subthemes addressing (1) the lack of smell or second hand smoke, (2) that e-cigarettes contain vapor and are not lit or burned, (3) that e-cigarettes do not have as many chemicals, and (4) that e-cigarettes can be used indoors were reasons given that they were thought to be a better alternative to smoking regular cigarettes.

— *“Cigarettes will kick up my asthma, e-cigs don’t”*
— *“They’re not supposed to have as many chemicals in them as the cigarettes do so maybe the cancer risk isn’t as high?”*
— *“I heard that there’s less chemicals in it”*
— *“It doesn’t smell bad—like it doesn’t seem—I don’t know if it’s bad for you but it doesn’t seem—it just doesn’t seem bad.”*
— *“The smoke doesn’t make so much damage to the others. And the nicotine is lower. A regular cigarette has more.”*
— *“I suppose it’s a safer alternative”*
— *“They say that they cause less damage than a normal cigarette.”*

### Electronic cigarette use in pregnancy

In order to assess the perceived risks of e-cigarette use during pregnancy, two different lead-in prompts were utilized. First, gravidae were asked, “What would you say to a pregnant friend who is using electronic cigarettes?” After they finished answering this question a follow-up prompt was asked: “What would you say to a pregnant friend who is using electronic cigarettes to quit smoking traditional cigarettes?” Although a common theme emerged in all focus groups that e-cigarettes are a safer alternative compared to regular cigarettes, when the discussion focused on their use during pregnancy, the major themes that emerged were that e-cigarettes are (1) not safe, (2) likely damaging to the baby, and (3) as bad as regular cigarettes during pregnancy. Minor themes which emerged were the suggestions to quit smoking either (4) cold turkey or (5) using the patch or gum. However, a contradicting minor theme which emerged was that e-cigarette use in pregnancy is not as bad as smoking if they are being used as a smoking cessation device. Because the focus groups were deidentified subjects and no personal information was collected, the current smoking status of participants was not known.

— *“I’d encourage her not to. It’s just as bad as a regular cigarette”*
— *“Smoking period is not good for you or your baby”*
— *“It’s not healthy for the baby”*
— *“It can cause problems to the baby’s forming”*
— *“They should think about the health of their kid”*
— *“I wouldn’t care. It’s just vapor, with flavor. As long as they don’t stink like cigarettes, I’m completely fine.”*
— *“I would be glad if she was smoking an electronic cigarette than just a regular cigarette.”*

### Stigma for electronic cigarette use in pregnancy

There is a well documented stigma against smoking combustible tobacco cigarettes in pregnancy [39, 40]. In order to determine if there is a similar stigma against e-cigarette use in pregnancy, two lead-in prompts were utilized. Women were asked, “What do you think when you see a pregnant woman smoking cigarettes?” followed by the question: “What would you think if you saw a pregnant woman using an electronic cigarette?” The women reported that smoking is not an acceptable option for pregnant women. When asked about smoking regular combustible tobacco cigarettes in pregnancy, participants said that (1) there are many health problems that can arise from smoking during pregnancy and (2) the behavior is selfish and irresponsible. When asked about the use of electronic cigarettes in pregnancy, facilitators noted that reactions were subjectively not as strong, but the women iterated their belief that there were still risks and therefore thought that the mother was not taking care of her baby’s health. However, a minor theme emerged that because there are fewer side effects, the mother might be using them to reduce the risks to her child.

***Smoking stigma:***

— *“It’s a sin to smoke during pregnancy.”*
— *“I think she’s harming her baby.”*
— *“I think she’s being selfish.”*
— *“I hope she cuts down.”*
— *“She doesn’t love the baby.”*

***Electronic cigarette use stigma:***

— *“You shouldn’t smoke anything.”*
— *“I would think the same (as about a pregnant women smoking regular cigarettes).”*
— *“It’s not healthy for the baby, you should avoid it.”*
— *“I mean I’m not for-sure what it is, but you know, there’s probably less side effects to it.”*
— *“I wouldn’t think nothing because I didn’t think it was harmful. That’s why I thought they were made—so, like, so we won’t get cancer, so people don’t have to smell it, so that’s why I thought they were actually better.”*

### Terminology and colloquialisms for e-cigarettes

To determine what words are colloquially being used to discuss and describe electronic cigarettes participants were asked, “What words or terms have you heard used to describe electronic cigarettes?” The main term utilized is “Blu,” which is a brand name of e-cigarettes. Other terms which were mentioned, but were minor themes were (1) e-cigs, (2) vaporizers and (3) smokeless/ smokefree.

### Knowledge of hookah

To assess baseline knowledge of perceived risks of hookah use in pregnancy, participants were asked “Tell me what you know about hookah.” Of note, hookah is known in the Hispanic community as “Pipa.” Although few Hispanic women knew what hookah was, translation to Pipa led to the majority of participants to engage in conversation. The two major themes which arose from discussion of hookah use were that (1) it is popular with teenagers and (2) hookah bars are a common place to use them. Other minor themes which arose included that (1) the tobacco comes in different flavors and (2) many have seen hookah being used on television programs such as reality shows and telenovelas.

— *“I know they have hookah bars”*
— *“…with a lot of teenagers”*
— *“And it attracts teenagers too”*
— *“I learned it on a TV show”*

### Hookah use during pregnancy

When asked “What would you say to a pregnant friend who is using hookah?” the major theme across all groups was unanimous. Women believed that hookah use was dangerous for the mother and baby. Of note, one participant had been using hookah until she found out she was 8 weeks pregnant. She immediately quit and was worried about the potential damage she had done to her fetus.

— *“I would educate that friend on the effects it has on her baby, their child, and the risk factor that they’re taking.”*
— *“It can mess up brain development.”*
— *“Don’t do it. Wait until after you have the baby.”*
— *“It harms the baby and also the mum’s health.”*
— *“I wouldn’t smoke hookah while pregnant, no sir.”*

## Discussion

Focus groups with pregnant women reveal that electronic cigarette or hookah use in pregnancy is perceived as posing health risks for the mother and fetus, with fewer perceived risks from electronic cigarette than regular cigarettes when used for smoking cessation. Electronic cigarette use among middle and high school students may quickly become a major public health issue. Because no studies are available on the long-term health effects of e-cigarettes, the consequences of use at such a young age will affect fertility and reproductive health is unknown. Furthermore, our current lack of understanding of the adverse effects of e-cigarette use in pregnancy on fetal development demonstrates a gap in our clinical knowledge. In this study we sought to elucidate, using a qualitative analysis, the perceived risks of e-cigarette and hookah use in pregnancy. We also sought to determine the common terms and colloquialisms used for e-cigarettes.

Overall, study participants were very knowledgeable about e-cigarettes. Because a focus group discussion format was used, women shared their knowledge of these devices from first or second-hand experience. Some women themselves had tried them, while others had spouses or family members who used them. While participants could explain where and how to buy them, they were much less knowledgeable about the ingredients and side effects. This is concordant with the current debate among regulatory agencies and health experts: there is a lack of disclosure of all ingredients in e-cigarettes on the part of manufacturers, and the health and safety claims lack validity [2]. They were knowledgeable about where they are sold, and had seen them advertised and used on television. Because these were gravidae, they asked questions about the safety of second hand vapor from e-cigarettes for their fetus. Specifically, what are the risks of spouses and family members using e-cigarettes in their presence, especially in closed spaces like inside a car? These are questions clinicians will face in the near future as e-cigarette prevalence and use continues to increase.

The overall impression of participants is that for non-pregnant individuals, e-cigarettes are a safer alternative to regular cigarette smoking. This may speak to the power of e-cigarette advertisements, as claims of harm reduction or benefits have not necessarily been required to be validated by clinical or scientific evidence. While studies of the safety and benefits of e-cigarettes compared to tobacco smoke are still few, some have shown that e-cigarettes may be beneficial as a harm reduction tool for current smokers [41–43]. Similarly, many participants felt that e-cigarettes are beneficial as smoking cessation devices for current smokers.

The strongest theme to emerge from our discussion on e-cigarette use in pregnancy is that women believed it is not safe for either the mother or fetus throughout gestation. However, a subtheme did emerge that it may not be a bad option to use them instead of smoking regular cigarettes while pregnant. We were also interested to determine whether there is a stigma for e-cigarette use in pregnancy. Lack of a stigma may influence a woman’s decision to use e-cigarettes during pregnancy as a smoking cessation device. While we did find many women believed that a pregnant woman using e-cigarettes is harming her baby, there were also women who thought e-cigarette use wasn’t as bad during pregnancy as regular cigarettes. Given the prevalent theme that e-cigarettes are a healthier alternative, and the increased acceptance of e-cigarettes in adolescents [3], it is possible that e-cigarette use in pregnancy will be less stigmatized than combustible tobacco cigarettes. As e-cigarettes become more prevalent, and younger people who started using them in middle and high school become pregnant, e-cigarette use may become more acceptable during pregnancy. One survey revealed that the perceptions of safety of e-cigarettes may increase their use during pregnancy [44]. With regard to hookah, focus group participants overwhelmingly agreed that these products are not safe to use in pregnancy, and questioned whether they were safe to use at any point.

While study participants disagreed with the use of any tobacco or nicotine products while pregnant, they overwhelmingly deferred all questions regarding safety to the woman’s doctor. This knowledge gap presents a potential opportunity for practitioners regarding how to help women quit smoking during pregnancy. A 2012 survey by the American College of Obstetrician and Gynecologists (ACOG) on screening practices and knowledge of obstetrician-gynecologists on emerging tobacco products revealed that only 5 % of respondents were fully informed about e-cigarettes, and 13.5 % believed they did not have any adverse effects on health [45]. However, it is our belief clinicians must be cautious. Although there are decades of research elucidating the danger of smoking combustible tobacco cigarettes, despite being widely available on the market since 2007, the safety of e-cigarette use, even in non-pregnant individuals, is still emerging in the literature.

While our study had significant participation and representation from all three clinics in order to achieve saturation, this study does have limitations. The focus groups were conducted using already established CenteringPregnancy® groups. These women were receiving comprehensive prenatal care including an hour of group discussions of healthy choices during pregnancy. Part of the CenteringPregnancy® model includes a discussion of tobacco use in pregnancy and strongly cautions against it. Therefore, these women had already been educated about the harmful side effects of combustible tobacco smoke. The attitudes and knowledge expressed by this group of women with access to comprehensive prenatal care may not be representative of populations with limited access to prenatal care or those who do not receive information about tobacco-related harm during their prenatal visits. In addition, because the groups were already established, it is possible that group dynamics were firmly established and influenced the women’s feedback and participation. We further note that because of the study design, we were not able to collect data on participant smoking status or demographics, and have a limited number of Caucasians in this subject population. A follow-up study by questionnaire, allowing an anonymous response, would enable us to collect the demographic and smoking status data, and would therefore help us gain quantitative information regarding e-cigarette use and the perception of risk.

## Conclusions

Clinicians can help their patients by asking them about their use of nicotine-containing products, including smoking, e-cigarettes and hookah. If gravidae are current smokers and are interested in quitting, e-cigarettes cannot currently be recommended as either a safe nor efficacious tool for smoking cessation. Thus, at present, the risk to benefit ratio is still being studied. An understanding of the health risks posed by e-cigarettes will help clinicians aid their patients to make truly informed decisions. Our qualitative study provides a population-wide reference cohort of gravidae with which to guide public health officials, patient advocacy groups, and physicians in the generation of knowledge and materials necessary for such gaps to be reliably closed. Until such a time, it is our belief that clinicians should inform their patients that abstinence from nicotine throughout gestation is advised.

## Abbreviations

E-cigarette: Electronic cigarette
